# What is known about human milk bank donors around the world: a systematic scoping review

**DOI:** 10.1017/S1368980021003979

**Published:** 2022-02

**Authors:** Bruna Gutierrez dos Santos, Maryanne T Perrin

**Affiliations:** Nutrition Department, University of North Carolina Greensboro, 319 College Avenue, 318 Stone Building, Greensboro, NC 27412, USA

**Keywords:** Milk banking, Donors, Human milk, Donor milk

## Abstract

**Objective:**

The WHO recommends that low birth weight infants receive donor human milk (DHM) when mother’s milk is not available. Systematic reviews have been published regarding clinical outcomes of infants receiving DHM, as well as the impact of pasteurisation on the composition of DHM; however, information about milk bank donors has not been systematically assessed.

**Design:**

We conducted a systematic scoping review of original research articles about milk bank donors published before August 2020.

**Setting:**

Globally.

**Participants:**

Donors to milk banks.

**Results:**

A total of twenty-eight studies were included across a variety of geographies: the USA (*n* 8), Brazil (*n* 7), Spain (*n* 4), India (*n* 2), and single studies in France, Norway, Poland, Italy, Taiwan, Korea and China. Study variables were grouped into six main categories: Donor Demographics (*n* 19), Clinical Characteristics (*n* 20), Donor Experiences (*n* 16), Donation Patterns (*n* 16), Lifestyle Characteristics (*n* 4) and Lactation/Breast-feeding History (*n* 8). Some demographic characteristics were commonly reported across regions, while other, including gender and race, were infrequently explored. Factors that might influence the composition of DHM, including birth timing (term or pre-term), milk type (colostrum, transition or mature) and maternal diet were not regularly studied. Other gaps in the literature included (1) donors’ motivations and barriers to donation, (2) lactation and breast-feeding history, including factors that influence donors to pump and amass surplus milk, and (3) donation patterns, including whether donors are also selling milk to corporations or sharing milk with peers.

**Conclusion:**

What is known about milk bank donors in different geographies is often limited to a single study, with heterogeneity in the variables reported.

The WHO recommends that low birth weight infants receive donor human milk (DHM) when mother’s own milk is not available due to evidence that it decrease the risk of necrotising enterocolitis^(1,2)^. Globally, DHM is typically produced by country-level milk banking networks that serve as a conduit between the recipient infants and the donors who provide the milk^(3–5)^. Although the recommended recipient for DHM is primarily the pre-term infant^(2,6)^, a recent review reported that DHM is also being used in other populations including healthy term infants and term infants with health risks. A 2020 report from a Virtual Communication Network of global milk banking leaders estimated that at least 800 000 infants receive DHM around the world annually^(7,8)^.

To ensure the quality and safety of DHM, human milk banks use similar hazard analysis and critical control points, where protocols are used in every step of the process, from donors screening until milk distribution^(9)^. Holder pasteurisation is the main processing technique used in milk banks, and although it inactivates virus such as HIV and cytomegalovirus, it also alters the milk composition^(10)^. A recent review found over forty studies that had evaluated the impact of Holder pasteurisation on DHM, suggesting that there is a growing body of knowledge about this technique^(10)^.

While there are multiple reviews on DHM recipients and milk banking processes, the donors to milk banks have not been systematically studied. A recent report by the WHO noted that ‘the motivations behind donating human milk remain under-researched’^(11)^. Other information about milk bank donors may provide important insights regarding donor recruitment and the nutritional care of infants receiving DHM. For example, a donor’s birth type (term *v*. pre-term) and milk type (colostrum, transition and mature) could influence the composition of the milk being collected by the milk banks^(12)^. Therefore, the aim of this review is to explore what is currently known about human milk bank donors globally and identify gaps for future research.

## Methods

A systematic scoping review was conducted to investigate what is known about milk bank donors. The objective of a scoping review is to map and summarise the information available for a research topic and to identify gaps where more research is needed^(13)^. The Preferred Reporting Items for Systematic Reviews and Meta-analysis (PRISMA) guidelines were used to guide this review. The databases used to identify original research articles were PubMed and Scopus. Search terms utilised for both databases included ‘Milk bank*’ AND ‘donors’ NOT (composition OR pasteuri* OR nutri*). Additional studies were located by hand-reviewing bibliographies of the studies identified through the primary search.

Original research articles about milk bank donors that were published before August 2020 were included in this review. Studies were excluded if they were (1) about donor milk composition and/or pasteurisation only, (2) about infant feeding practices and/or infant nutrition only, (3) in languages that were not English, (4) not original articles or (5) not about milk bank donors (e.g. peer-milk sharing only). Two researchers (BGS and MTP) independently evaluated all study titles, abstracts, and full papers for exclusion or inclusion criteria, and differences were resolved after each review step by discussion.

Included studies were independently abstracted by two researchers (BGS and MTP) into a Microsoft Excel spreadsheet for the following information: study location, study design, study population, study objectives, data collection methods, variables related to milk bank donors, results and funding source. Studies that used multiple years of milk bank donor data were classified as semi-longitudinal study design, since some donors may have appeared more than once in data that spanned several years. Abstracted data were reviewed by two researchers (BGS and MTP) and discrepancies were resolved by discussion. Demographic data from one study combined donor and non-donor information and could not be interpreted; therefore, these demographic data were not reported in the results.

To organise study variables, an iterative process was used by two researchers working together to develop and refine a classification system of main categories and sub-categories for study variables. Categories and sub-categories used to classify variables included (1) Donor Demographics (Demographics) which included Age, Marital Status, Race-Ethnicity, Education, and Employment Status, (2) Donor Clinical Characteristics (Clinical) which included Birth History (e.g. number of children, parity, delivery term, neonatal intensive care unit (NICU) admissions), Diseases (e.g. donor health conditions) and Prenatal Care, (3) Donor Lifestyle Characteristics (Lifestyle) which included Diet, Exercise, Legal Drug Use (e.g. nicotine, caffeine, and alcohol) and Illegal Drug Use, (4) Lactation and Breast-feeding Experience (Breast-feeding) which included Breast-feeding History (e.g. breast-feeding experience and problems), Clinical Support, Milk Expression Practices, and Beliefs About the Value of Milk, (5) Donor Experience and Beliefs (Experience/Beliefs) included Reasons/Enablers for Donation, Barriers for Donation and Donor Identity and (6) Donation Patterns (Patterns) included Donation Volume, Donor Type (first-time or repeat), Milk Type (colostrum – 0–7 d, transition milk – 7–21 d, mature milk – over 21 d)^(14)^, and Donation Duration.

The primary source of bias considered was selection bias, if donors included in a study were potentially not representative of the broader donor population. Studies were identified as possibly having selection bias if they did not discuss participant selection, had low participation rate (below 60 %)^(15)^ or included a limited sampling frame (e.g. only bereaved donors, only donors active on social media). Selection bias was evaluated independently by two researchers and discrepancies were resolved by discussion.

## Results

**Fig. 1:**
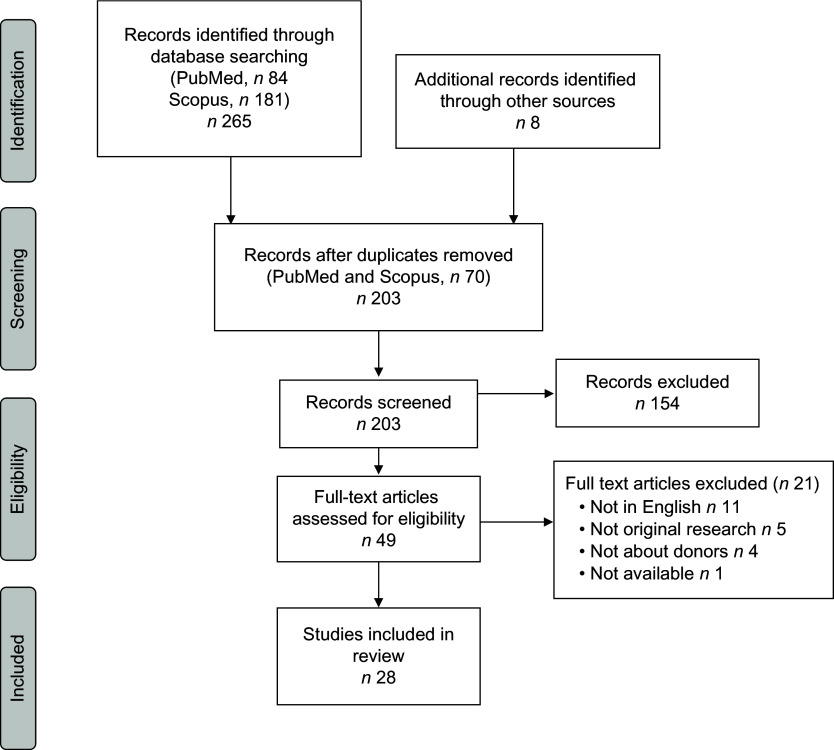
Flow diagram of the literature search process used to identify studies using the Preferred Reporting Items for Systematic Reviews and Meta-Analyses (PRISMA) checklist

A total of 181 studies were identified through Scopus, 84 through PubMed and 8 through hand-review of bibliographies (Fig. 1). After excluding duplicates (*n* 70), a total of 203 studies were screened. After a review of abstracts and titles, 154 articles were excluded leaving 49 articles for full-text review. Twenty-one studies were excluded after full-text review leaving twenty-eight studies in this scoping review about human milk bank donors^(16–43)^.

Studies in this systematic review were published between 2003 and 2020 (Table 1) and included 2 to 4000 donors. Eight studies were conducted in the USA, seven in Brazil, four in Spain, two in India, and individual studies were conducted in France, Norway, Poland, Italy, Taiwan, Korea and China. A qualitative design was used in eight studies, which allows for rich exploration of the donors’ lived experiences. Qualitative studies were predominantly conducted in the USA and had a small sample size (2–21 donors and 80–107 online testimonials or images). Data collection methods used in the studies included interviews, questionnaires, chart reviews and online content analysis. In most of the studies, donors were recruited from a single milk bank (*n* 16). Ten studies (36 %) presented possible selection bias (Table 1). The number of studies reporting variable types included (1) Donor Demographics (*n* 19; Table 2), Clinical Characteristics (*n* 20; Table 3), (3) Lifestyle Characteristics (*n* 4; Table 4), (4) Lactation/Breast-feeding Experiences (*n* 8; Table 5), (5) Donor Experiences (*n* 16; Table 6) and (6) Donation Patterns (*n* 16; Table 7).

**Table 1:** Summary of studies included in the systematic scoping review of human milk bank donors

Year	Author	Study location	Study objectives	Population studied	Study design	Data collection method	Potential selection bias	Funding source
2003	Azema^(16)^	France	Examine characteristics of donors and attitudes towards donation	Donors to eight milk banks (*n* 103)	Cross-sectional	Questionnaire		Information not available
2004	Lindemann^(17)^	Norway	Evaluate donor characteristics and donation patterns	Donors to a single milk bank in 2001 (*n* 69)	Not identified	Not identified		Information not available
2007	Osbaldiston^(18)^	USA	Compare donors and non-donors characteristics, experiences, motives and barriers to donation, and the relationship between donation experience and amount of milk donated	Donors to a single milk bank (*n* 87) and non-donor controls (*n* 19)	Case–control	Telephone survey that included VFI, PANAS, scale questions; chart review	Possible	Information not available
2008	Thomaz^(19)^	Brazil	Identify factors that influenced or motivated donations	Donors to three milk banks (*n* 737)	Cross-sectional	Questionnaire		Information not available
2009	Alencar^(20)^	Brazil	Describe the behaviour, beliefs and feelings behind the donations	Donors to two milk banks (*n* 36)	Cross-sectional	Structured and semi-structured face-to-face interviews		Information not available
2010	Alencar^(21)^	Brazil	Characterise the behaviour of donation and formal/informal support	Donors to two milk banks (*n* 36)	Cross-sectional	Structured and semi-structured face-to-face interviews		Information not available
2010	Cohen^(22)^	USA	Estimate the seroprevalence of hepatitis B and C, syphilis, HTLV-1 and 2 and HIV	Potential donors to a single milk bank from 2000 to 2005 (*n* 1091)	Semi-longitudinal	Chart review		Information not available
2010	Koyashiki^(23)^	Brazil	Evaluate the degree of exposure to lead of donors	Donors to a single milk bank (*n* 92)	Cross-sectional	Face-to-face interview, questionnaire, milk sample, blood sample		Information not available
2012	Welborn^(24)^	USA	Examine the role of milk donation in the grieving process	Bereaved donors to two milk banks (*n* 21)	Qualitative, phenomenological	Semi-structured face-to-face and web-based interviews	Possible	No funding obtained
2013	Chang^(25)^	Taiwan	Evaluate donor characteristics and donation patterns	Donors to a single milk bank from 2005–2010 (*n* 816)	Semi-longitudinal	Chart review		Information not available
2013	Pineau^(26)^	USA	Describe how intensive motherhood and social class influence milk donations	Donors to a single milk bank (*n* 19), including sixteen middle/upper income and three WIC recipients	Qualitative	Face-to-face and telephone interviews	Possible	Information not available
2014	Escuder-Vieco^(27)^	Spain	Validate the health questionnaire with respect to the presence of illegal drugs, nicotine and caffeine in donor milk	Donors to a single milk bank (*n* 63)	Cross-sectional	Questionnaire and milk samples		Spanish Health Research Funding
2014	Sierra-Colomina^(28)^	Spain	Compare the donors social and demographic characteristics with the volume of milk donated	Donors to a single milk bank from 2009–2013 (*n* 391)	Semi-longitudinal	Questionnaire and chart review		SAMID (Spanish Collaborative Maternal and Children and Development) Research Network
2015	Machado^(30)^	Spain	Describe experiences, beliefs, motivations and difficulties of donations	Donors to a single milk bank (*n* 7)	Qualitative phenomenological	Semi-structured interviews	Possible	Information not available
2016	Escuder-Vieco^(29)^	Spain	Determine levels of illegal drugs, nicotine and caffeine in hair and breast milk	Donors to a single milk bank (*n* 36)	Cross-sectional	Questionnaire; hair and milk samples		Spanish Health Research Funding
2016	Jang^(31)^	Korea	Evaluate donor characteristics and donation patterns	Donors to a single milk bank from 2008–2015 (*n* 915)	Semi-longitudinal	Chart review using standardised form		Information not available
2016	Miranda^(32)^	Brazil	Investigate milk donor’s representations of the donation experience	Donors to a single milk bank (*n* 12)	Qualitative	Semi-structured interview	Possible	Universidade Federal de Ouro Preto
2017	Barbarska^(33)^	Poland	Evaluate donor characteristics and donation patterns	Donors to a single milk bank from 2015–2016 (*n* 45)	Semi-longitudinal	Chart review		Information not available
2017	Kupek^(34)^	Brazil	Estimate the seroprevalence of HIV, syphilis and hepatitis B	Prospective donors to a single milk bank from 2005–2015 (*n* 3513)	Semi-longitudinal	Chart review		No funding obtained
2017	Meneses^(35)^	Brazil	Estimate prevalence and factors associated with donation	Donors to nine milk banks (*n* 51) and non-donors control (*n* 644)	Case–control	Structured interviews	Possible	Fundação de Amparo à Pesquisa do Estado do Rio de Janeiro – FAPERJ
2018	Candelaria^(36)^	USA	Examine donors’ experiences donating to milk banks	Donors with infants in the NICU (*n* 12)	Qualitative phenomenological	Questionnaire and semi-structured face-to-face interviews	Possible	No funding obtained
2018	Cole^(37)^	USA	Examine milk donation in the context of perinatal palliative care	Bereaved donors (*n* 2)	Qualitative case study	Questionnaire and telephone interview	Possible	No funding obtained
2018	Quitadamo^(38)^	Italy	Describe donation volume by donor clinical characteristics	Donors to a single milk bank from 2010–2017 (*n* 659)	Semi-longitudinal	Chart review		Information not available
2019	Liu^(39)^	China	Characterise milk bank donors and donation patterns	Donors to fourteen milk banks 2013–2016 (*n* 2680)	Semi-longitudinal	Chart review		Guangdong provincial commission of health and family planning appropriate technology promotion project (2015–2017 Guangdong)
2019	Oreg^(40)^	USA	Explore milk donation in times of loss to uncover mechanisms liking grief and loss to philanthropic giving	Bereaved donors (*n* 80)	Qualitative phenomenological	Content analysis of online testimonials	Possible	Information not available
2019	Sachdeva^(41)^	India	Evaluate the status of milk banks	Donors to sixteen milk banks from 2015 to 2016 (range 70–4000 per bank)	Semi-longitudinal	Online questionnaire and on-site interview of milk bank personnel		Margaret A. Cargill Philanthropies to PATH
2020	Nangia^(42)^	India	Classify donors by demographics; determine and compare milk volume donated by donor classifications.	Donors to a hospital milk bank from 2017–2019 (*n* 1553)	Semi-longitudinal	Chart review		No funding obtained
2020	Oreg^(43)^	USA	Determine characteristics of the milk donor identity	Donors’ online testimonial (*n* 95) and images (*n* 107)	Qualitative phenomenological	Content analysis of online donor testimonials and images	Possible	Information not available

VFI, volunteer functions inventory; PANAS, positive and negative affect schedule; WIC, Women, Infants, and Children programme; NICU, neonatal intensive care unit.

**Table 2:** Demographic information about milk bank donors

Sub-category	Country	Year	Subjects	Findings
Age (years)	Brazil^(19)^	2008	737 donors	Majority < 25 (18 % < 18; 41 % 18 to 24)
	Brazil^(20,21)^	2009, 2010	36 donors	Ranged from 14 to 33; mean age 25
	Brazil^(23)^	2010	92 donors	Ranged from 16 to 45; mean age 21
	Brazil^(32)^	2016	12 donors	Ranged from 18 to 39; mean age 26
	Brazil^(34)^	2017	3513 donors	Majority 20 to 35 (80 %)
	China^(39)^	2019	2680 donors	Majority 25 to 35 (82 %); mean age 29
	France^(16)^	2003	103 donors	Ranged from 20 to 42; mean age 31
	India^(42)^	2020	1553 donors	Majority < 25 (88 %)
	Korea^(31)^	2016	915 donors	Majority 30 to 39 (70 %)
	Norway^(17)^	2004	69 donors	Ranged from 21 to 45; mean age 34
	Poland^(33)^	2017	45 donors	Ranged from 23 to 44; mean age 32
	Spain^(28)^	2014	391 donors	Median age of 34; IQR of 31–36
	Spain^(27)^	2014	63 donors	Ranged from 23 to 53; mean age 36
	Spain^(30)^	2015	7 donors	Ranged from 21 to 39; mean age 32
	Spain^(29)^	2016	36 donors	Ranged from 24 to 41; mean age 34
	Taiwan^(25)^	2013	816 donors	Ranged from 18 to 45; mean age 31
	USA^(18)^	2007	87 donors	Majority 30–39 (73 %)
	USA^(36)^	2018	12 donors	All < 40 (50 % 21–29; 50 % 30–39)
Marital status	Brazil^(19)^	2008	737 donors	Single (54 %)
	Brazil^(20)^	2009	36 donors	Married or in a partnership (78 %)
	Brazil^(32)^	2016	12 donors	Married or in a partnership (75 %)
	France^(16)^	2003	103 donors	Married or in a partnership (97 %)
	Spain^(30)^	2015	7 donors	Married (86 %)
	USA^(18)^	2007	87 donors	Married (91 %)
	USA^(36)^	2018	12 donors	Married (100 %)
Race-ethnicity	Brazil^(23)^	2010	92 donors	White (72 %)
	USA^(18)^	2007	87 donors	White (87 %)
	USA^(36)^	2018	12 donors	White (100 %)
Education	Brazil^(19)^	2008	737 donors	Some college/higher education (5 %)
	Brazil^(20)^	2009	36 donors	Some college/higher education (36 %)
	Brazil^(23)^	2010	92 donors	Some college/higher education (48 %)
	Brazil^(32)^	2016	12 donors	Completed high school (92 %)
	China^(39)^	2019	2680 donors	College/higher education (60 %)
	Norway^(17)^	2004	69 donors	College/higher education (73 %)
	Spain^(30)^	2015	7 donors	College/higher education (majority)
	Taiwan^(25)^	2013	816 donors	College/higher education (81 %)
	USA^(18)^	2007	87 donors	College/higher education (83 %)
Employment status	Brazil^(19)^	2008	737 donors	Unemployed (70 %)
	Brazil^(20)^	2009	36 donors	Worked outside the home (47 %)
	Brazil^(32)^	2016	12 donors	Housewives (42 %)
	China^(39)^	2019	2680 donors	Worked outside the home (85 %)
	France^(16)^	2003	103 donors	Worked outside the home (51 %)
	Korea^(31)^	2016	915 donors	Housewives (62 %)
	Spain^(30)^	2015	7 donors	Worked outside the home (majority)
	Taiwan^(25)^	2013	816 donors	Worked outside the home (72 %)
	USA^(18)^	2007	87 donors	Worked outside the home (65 %)

**Table 3:** Clinical information about milk bank donors

Sub-category	Country	Year	Subjects	Finding
Birth history	Brazil^(19)^	2008	737 donors	Delivered pre-term (47 %); had < 3 children (94 %)
	Brazil^(20)^	2009	36 donors	Had 1 child (61 %)
	Brazil^(23)^	2010	92 donors	Had 1 child (67 %)
	Brazil^(32)^	2016	12 donors	Primiparous (83 %)
	Brazil^(34)^	2017	3513 donors	Multiparous (94 %)
	Brazil^(35)^	2017	51 donors; 644 non-donors	Donors less likely to have infant in NICU than non-donors
	China^(39)^	2019	2680 donors	Delivered pre-term (8 %)
	France^(16)^	2003	103 donors	Had 1 to 2 children (83 %)
	India^(42)^	2020	1553 donors	Delivered pre-term (53 %); multiparous (57 %); infant admitted to NICU (37 %)
	Italy^(38)^	2018	659 donors	Delivered after 35 weeks of gestational age (94 %)
	Norway^(17)^	2004	69 donors	Most donors were primiparous and delivered at term (% not provided)
	Poland^(33)^	2017	45 donors	Delivered pre-term (24 %)
	Spain^(27)^	2014	63 donors	Delivered pre-term (21 %); primiparous (62 %)
	Spain^(28)^	2014	391 donors	Delivered pre-term (23 %); primiparous (56 %); infant admitted to NICU (37 %)
	Spain^(30)^	2015	7 donors	Had 1 to 2 children (100 %)
	Spain^(29)^	2016	36 donors	Delivered pre-term (17 %)
	Taiwan^(25)^	2013	816 donors	Delivered pre-term (8 %); primiparous (69 %)
	USA^(18)^	2007	87 donors	Had 1 to 2 children (80 %)
	USA^(36)^	2018	12 donors	Primiparous (50 %); had infant in NICU (100 %)
Disease	Brazil^(34)^	2017	3513 donors	HIV prevalence decreased to 0 %, syphilis increased to 1·8 %, and acute hepatitis B increased to 3 % over 10 years.
	Poland^(33)^	2017	45 donors	Had chronic disease not contraindicated to donation (24 %)
	USA^(22)^	2010	1091 donors	3·3 % rejected for abnormal serological screening
Prenatal care	Brazil^(20)^	2009	36 donors	Attended 3–30 prenatal healthcare visits (100 %)
	Brazil^(32)^	2016	12 donors	Attended 7–12 prenatal healthcare visits (100 %)

NICU, neonatal intensive care unit.

**Table 4:** Lifestyle characteristic information about milk bank donors

Sub-category	Country	Year	Subjects	Finding
Diet	USA^(18)^	2007	87 donors	Self-reported always/nearly always eating healthy food (56 %)
Exercise	USA^(18)^	2007	87 donors	Self-reported exercising 3+ times/week (64 %)
Legal drug use	Brazil^(23)^	2010	92 donors	Self-reported never having smoked (82 %)
	USA^(18)^	2007	87 donors	Self-reported alcohol consumption < 1 time/month (77 %)
	Spain^(27)^	2014	63 donors	Presence of caffeine (45 % of milk samples); presence of nicotine (0·3 % of milk samples)
	Spain^(29)^	2016	36 donors	Presence of caffeine (50 % of milk and 78 % of hair samples); presence of nicotine (0 % of milk and 3 % of hair samples at threshold of active smoker)
Illegal drug use	Spain^(27)^	2014	63 donors	Presence of illegal drugs (0 % of milk samples)
	Spain^(29)^	2016	36 donors	Presence of illegal drugs (0 % of milk and 0 % of hair samples)

**Table 5:** Lactation and breast-feeding experience information about milk bank donors

Sub-category	Country	Year	Subjects	Finding
Breast-feeding history	France^(16)^	2003	103 donors	Excellent/good breast-feeding experience (97 %);
	USA^(36)^	2018	12 donors	Exclusive breast-feeding (100 %)
Clinical support	Brazil^(35)^	2017	51 donors; 644 non-donors	Clinical support associated with being a donor included (1) receiving in-hospital help with breast-feeding and (2) receiving information about milk expression
Milk expression practices	Brazil^(21)^	2010	36 donors	Expressed manually (61 %); expressed milk 1+ times/d (72 %); factors influencing expression included beliefs about impact of diet (47 %), availability of time (28 %) and negative emotions (28 %).
	USA^(18)^	2007	87 donors; 19 non-donors	Expressed with personal electrical pump (75 %); donors reports fewer problems with pumping than non-donors
Beliefs about the value of milk	Brazil^(32)^	2016	12 donors	Major theme: importance of breast-feeding for both the baby and the mother
	Spain^(30)^	2015	7 donors	Major theme: benefits of breast-feeding
	USA^(26)^	2013	19 donors	Major themes: breast milk being a cure for everything, a gift with expiration date, majority of middle- and upper-income donors expressed an interest of receiving compensation

**Table 6:** Donor experience information about milk bank donors

Sub-category	Country	Year	Subjects	Findings
Reasons/enablers to donation	Brazil^(19)^	2008	737 donors	Encouraged by a health professional (61 %), received information in the hospital (50 %)
	Brazil^(20)^	2009	36 donors	Altruism (92 %), excess milk production (61 %), to avoid waste (47 %), information provided by healthcare professionals and media (47 %)
	Brazil^(21)^	2010	36 donors	Received support from family (89 %) and institution (58 %)
	Brazil^(32)^	2016	12 donors	Major themes: altruism, avoid waste, institutional and family support
	Brazil^(35)^	2017	51 donors; 644 non-donors	Donors were significantly more likely to be encouraged to donate milk at the hospital than non-donors
	China^(39)^	2019	2680 donors	The internet was the most popular source of information regarding donations (33 %)
	France^(16)^	2003	103 donors	Having excess milk (57 %) and desire to help others (41 %)
	Korea^(31)^	2016	915 donors	Obtained information about donation online (76 %)
	Spain^(30)^	2015	7 donors	Major themes: information received about milk banks and perceived approval of family and friends, having excess milk, altruism, empathy, support from family and milk bank
	USA^(18)^	2007	87 donors	To help others, having excess milk (% not provided)
	USA^(24)^	2012	21 donors	Major themes: physical and emotional meanings of pumping, finding meaning in perinatal loss, and importance of healthcare providers addressing lactation with bereaved mothers
	USA^(26)^	2013	19 donors	Major theme: deriving value from the physical and emotional labour of pumping
	USA^(36)^	2018	12 donors	Major themes: hope of donation helping others, act of donating was nurturing for the donor, importance of support from healthcare staff and desire to share their stories
	USA^(37)^	2018	2 donors	Major themes: milk donation as a mean of processing perinatal loss and doing something helpful with their milk
	USA^(43)^	2020	95 donor testimonials	Major theme: having excess milk
Barriers for donation	Brazil^(20)^	2009	36 donors	Main reasons to cease donation included returning to work and reduction in milk production
	Brazil^(32)^	2016	12 donors	Major theme: limited information provided prenatally
	Spain^(30)^	2015	7 donors	Major themes: lack of healthcare provider knowledge, distance from milk bank, no support at work and decrease of milk production
	USA^(18)^	2007	87 donors	Finding time to pump, transporting milk to the bank and problems getting blood test (% not provided)
	USA^(37)^	2018	2 donors	Major theme: frequent pumping was difficult
Donor identity	USA^(24)^	2012	21 donors	Major themes: identifying as a bereaved mother/grieving the loss of motherhood
	USA^(40)^	2019	80 donors	Major themes: a temporal donor identity allowed bereaved mothers opportunity to process loss and reconstruct maternal/female identity
	USA^(43)^	2020	95 donor testimonials	Major themes: donors had complex and fluid identity including being a woman, a mother, healthcare professional and prior recipient of milk donation

**Table 7:** Donation pattern information about milk bank donor

Sub-category	Country	Year	Subjects	Findings
Donation volume	China^(39)^	2019	2680 donors	1·9 l (mean)
	India^(41)^	2019	70–4000 donors	0·64 l (median)
	India^(42)^	2020	1553 donors	0·27 l (mean); significantly higher volumes were donated by mothers with infants in the NICU *v*. postnatal wards
	Italy^(38)^	2018	659 donors	2·9 l (mean) for term donors and 11·7 l (mean) for pre-term donors
	Korea^(31)^	2016	915 donors	11·8 l (mean)
	Norway^(17)^	2004	69 donors	29 l (mean)
	Poland^(33)^	2017	45 donors	0·65–32 l (range)
	Spain^(28)^	2014	391 donors	3·1 l (median), 0·04–174 l (range); donation volume was significantly higher with donors whose infants were hospitalised, had lower gestational age at birth, lower infant age at time of donation and were previously milk bank donors
	Taiwan^(25)^	2013	816 donors	17 l (mean)
	USA^(18)^	2007	87 donors	30 l (mean)
Donor type	Brazil^(20)^	2009	36 donors	First-time donors (83 %)
	Brazil^(32)^	2016	12 donors	First-time donors (92 %)
	China^(39)^	2019	2680 donors	Repeat donors (donated more than three times) (55 %)
	France^(16)^	2003	103 donors	First-time donors (72 %)
	Korea^(31)^	2016	915 donors	First-time donors (51 %)
	Taiwan^(25)^	2013	816 donors	First-time donors (97 %)
Milk type	Brazil^(20)^	2009	36 donors	Started donating within 3 weeks after delivery (colostrum/transition milk) (47 %)
	Brazil^(23)^	2010	92 donors	Majority of donations were mature milk (83 %)
	China^(39)^	2019	2680 donors	Started donating after 1 month postpartum (77 %) (mature milk)
	Korea^(31)^	2016	915 donors	Majority of donations were from 1 to 3 months postpartum (mature milk)
	Norway^(17)^	2004	69 donors	Started donating on average when infant was 7 weeks old. Range of infant age at start was 1–21 weeks (transition and mature milk)
	Poland^(33)^	2017	45 donors	Started donating on average when infant was 14 weeks old. Range of infant age at start was 1–44 weeks (transition and mature milk)
	Spain^(28)^	2014	391 donors	Started donating on average when infant was 12 weeks old. Range of infant age at start was 0–28 months old (colostrum to mature milk)
	Spain^(27)^	2014	63 donors	Majority of donations were mature milk (91 %)
	Taiwan^(25)^	2013	816 donors	Majority of donors (97 %) began donating > 1 month postpartum (mature milk)
Donation duration	Brazil^(20)^	2009	36 donors	From 1 to 4 months
	Norway^(17)^	2004	69 donors	From <1 to 13 months
	Poland^(33)^	2017	45 donors	From 2–26 weeks
	USA^(37)^	2018	2 donors	From 6–8 weeks

NICU, neonatal intensive care unit.

## Discussion

Despite reports that there are now over 600 milk banks operating around the world^(44)^, and over 800 000 infants annually who receive DHM^(7)^, studies about milk bank donors are often limited to a single study per geography with significant heterogeneity in the variables reported.

### Donor demographics

Age was the most commonly reported demographic variable, with some initial geographic differences observed. Specifically, donors were predominantly in their early- to mid-twenties in Brazil and India (based on mean donor age or prevalence of donors by age group)^(20,21,23,32,34,42)^, while donors were predominantly in their early-thirties in France, Korea, Norway, Poland, Spain, Taiwan and the USA^(16–18,25,27–31,33)^. There were also geographic differences in education levels among donors, with studies conducted in Brazil reporting that the majority of donors were not college-educated compared to mostly college-educated donors in China, Norway, Spain, Taiwan and the USA^(17,19,20,23,25,25,30,39)^. Across all geographies, donors were predominantly married or living with a partner^(16,18–20,30,32,36)^. Limited information was available on race-ethnicity^(18,23,36)^. No information was collected about gender in any of the studies, suggesting that donor gender may have been assumed in prior research. While this scoping review identified some differences in donor demographics across geographies, interpretation of this information requires more context related to the local setting.

### Donor clinical characteristics

Birth history frequently included a donor’s number of children. Results varied by geographies, with some studies reporting that donors were predominantly primiparous and others predominantly multiparous^(16–20,23,25,27–30,32–36,38,39,42)^. The percentage of donors that had pre-term births were in the minority in most studies (8–24 %)^(25,27–29,33,39)^, though two studies in India and Brazil reported the approximately half of donors gave birth pre-term^(19,42)^. Donor birth term could influence the composition of some nutrients in donor milk if donations are made in the first weeks postpartum^(45,46)^, suggesting that this may be useful donor data to regularly collect. Information regarding donors’ diseases/conditions^(22,33,34)^ and prenatal clinical care was limited^(20,32)^. Data on characteristics of the donor’s child beyond birth term were also scarce. For example, no studies reported the sex of the donor’s infant, and only a few studies reported hospitalisation status.

### Donor lifestyle characteristics

There is limited research regarding donors’ lifestyle characteristics including diet, exercise, legal and illegal drug use, which does not allow for any type of synthesis across regions. While milk banks screen donors to ensure they are healthy, lifestyle information could be valuable, as factors associated with maternal diet and lifestyle may influence what is being transferred in the milk.

### Lactation and breast-feeding experience

Donors reported similar beliefs about the importance of breast-feeding and breast milk across three geographies^(26,30,32)^. Donors’ beliefs in the value of their milk was only explored in one study, with many donors expressing the desire for compensation. Information about donors’ breast-feeding history, clinical support for lactation and milk expression practices was limited to one or two studies, suggesting this is an important area for future research to better understand the donor’s path to having excess milk for donation.

### Donor Experiences and Beliefs

The most common donor experience studied was reasons/enablers for donation^(16,18–21,24,26,30–32,35,37,39,43)^. Common reasons for donation included altruism, having excess milk and avoiding waste^(16,18,20,30,32,36,37,43)^. Common enablers for donation were being encouraged to donate and receiving information about milk banks from healthcare providers^(19–21,24,30–32,35,36,39)^. Healthcare providers were reported as a major source of information in Brazil, while online sources were reported as major sources of information in Korea and China^(19–21,31,39)^. Barriers for donation were only assessed in three countries and included finding time to pump, reduced milk production, limited information provided prenatally, returning to work, distance from milk bank and no support at work^(18,20,30,32,37)^. Qualitative studies that explored donor identity were all conducted in the USA and found that while the act of donating influenced mother’s identity, it had a special meaning for bereaved mothers^(24,37,40)^.

### Donation patterns

There was a wide range of reported donation volumes per donor (mean or median 0·64–30 l and range 0·04–174 l)^(17,18,25,28,31,33,38,39,41,42)^. The wide range could be attributed to the differences in milk banking requirements. For example, in Brazil, there is not a minimum donation volume^(47)^, while in the USA some milk banks require a minimum donation of 100 ounces^(48)^. In India and Spain, donors with infants in the NICU/hospitalised provided significantly higher volumes than donors without hospitalised infants^(28,42)^. Donor type was mostly first time (*v*. repeat) in all regions, although it was not widely reported^(16,20,25,31,32)^. The type of milk commonly donated was mature milk, as the donations started mostly after 1 month postpartum^(17,20,25,27,28,31,33,39)^. This suggests that donors are frequently providing milk that is likely lower in protein than the colostrum and transition milk that would normally be provided by an infant’s own mother in the early postpartum period. There was limited information about donation duration (range 2 weeks to 13 months)^(17,20,33,37)^. No studies collected information regarding whether milk bank donors provided their milk elsewhere, including either selling it or sharing with a peer.

## Conclusion and future direction

Although DHM banking continues to grow around the world^(49,50)^, information about the individuals who donate their milk is often limited to a single study per geography, with heterogeneity in the variables reported. Further, one-third of the studies were subject to potential selection bias. Some demographic characteristics were commonly reported across regions, while others, including gender and race, were infrequently explored, suggesting the need to incorporate these demographic variables in future research. Although donors’ experiences related to donations were frequently reported, enablers and barriers for donation differ among regions studied and not enough is known about what motivates donors to donate. Additionally, factors that could influence the nutritional profile of DHM, including birth timing (term or pre-term), type of milk donated (colostrum, transition or mature), donor diet and infant characteristics, should be more frequently collected. Other factors that have not been widely studied included donor lactation and breast-feeding history, including factors that influence why donors are pumping and amassing surplus milk and donation patterns, including whether milk bank donors are also selling milk to corporations or sharing milk with peers.
